# Global Innovation Trends for Plant-Based Vaccines Production: A Patent Analysis

**DOI:** 10.3390/plants10122558

**Published:** 2021-11-23

**Authors:** Dario G. Frisio, Vera Ventura

**Affiliations:** 1 Department of Environmental Science and Policy, Università degli Studi di Milano, 20133 Milano, Italy; dario.frisio@unimi.it; 2 Department of Civil, Environmental, Architectural Engineering and Mathematics, University of Brescia, 25133 Brescia, Italy

**Keywords:** innovation, patents, COVID-19, plant-based vaccines, biopharma

## Abstract

The use of plants as biofactories for the production of medical products and vaccines has a long history, but the recent COVID-19 pandemic has caused this set of technologies, for their potential to contribute to the development of innovative solutions for tackling pandemic spread worldwide, to rise in prominence. The purpose of this paper is to analyze the global innovation scenario of plant-based vaccine production. Methods: Patent search using a specific set of technical classification codes and keywords was performed using the Questel-Orbit database, with a final output of 180 patent families, corresponding to 1397 single patents. Results: Plant-based vaccines production is an innovation sector with positive development especially in the last five-year period (30% growth). Fifty percent of the patents were registered in the United States, standing out as the most attractive patent system worldwide. The inventive activity was led by private firms owning the 49% of the patent families, and the key-players group includes the companies that successfully developed plant-based COVID-19 vaccine candidates, indicating a strong connection between the expertise in innovation production and the capacity to adapt inventions to the current pandemic vaccine demand. Virus-like particles technology has increased in importance over the past few years. Conclusion: Patent data confirm their relevant role as indicators of innovation and technological evolution. Plant-based vaccines are expected to acquire an increasing role over the next few years as the current pandemic acts as an innovation catalyst.

## 1. Introduction

The recent COVID-19 pandemic vigorously raised the issue of vaccine production and availability with extreme urgency because, despite the development of several effective vaccines, new products are required to supply the increasing demand. In addition to the vaccines produced with traditional pharmaceutical technologies already on the market, researchers worldwide are working on new vaccine candidates with different technologies and are at different stages of development [1]. Among them, plant-based vaccines have acquired increased attention as seven vaccine candidates have been already developed and are currently under evaluation: the most advanced in the trial phase is the one developed by the Canadian biopharmaceutical company Medicago (owned by a subsidiary of Mitsubishi Chemical Corporation (67%) and Phillip Morris International) in collaboration with GlaxoSmithKline, which is produced in a wild tobacco relative (Nicotiana benthamiana) used as a biofactory to obtain virus-like particles against COVID-19. The available published results of the phase 1 randomized trials outlined low adverse effects and significant antibody response [2]. Moreover, the company iBio Inc. is working on a plant-based vaccine candidate designed to overcome the current challenges of first-generation vaccines (durability, access, and variant inclusion) and is currently in the pre-clinical trial phase. A plant-based vaccine candidate developed by Kentucky BioProcessing (owned by British American Tobacco) company with the potential to be stable at room temperature (a significant advantage for healthcare systems) is to date in phase 1/2 of clinical trial [3,4], while a further plant-based vaccine candidate (phase 1) is owned by a spin-off company from the faculty of pharmaceutical sciences Chulalongkorn University (Thailand).

More recently, four new products entered the pre-clinical trial phase, as shown in Table 1, confirming the huge interest towards plant-based solutions for vaccine production (The updated list of COVID-19 vaccine candidates can be found at https://www.who.int/publications/m/item/draft-landscape-of-COVID-19-candidate-vaccines, accessed on 3 November 2021). Nevertheless, long before the COVID-19 pandemic outbreak, the research for plant-based vaccines targeting different infectious diseases had already led to the development of several commercial products, such as the treatment for Ebola produced in tobacco plants by the firm Kentucky BioProcessing in 2014, or plant-based vaccines for veterinary use, such as a vaccine against the Newcastle disease for poultry developed by the USDA Center for Veterinary Biologics and approved in 2006 [5].

Vaccine production in plants is part of a broad branch of technologies that enable the production of a wide range of compounds (antibodies, and drugs) using plants as biofactories, including for cancer therapies [6] and hepatitis B [7,8]. Moreover, in general, the use of plants to produce biomolecules useful to humans is an innovative area of research that had already started at the beginning of the 1990s under the name of biopharming, or plant molecular farming, intended as the use of genetically modified plants to produce a wide range of pharmaceuticals and industrial products [9,10,11]. From an economic perspective, the main advantage of plant molecular farming was the rapid and scalable supply of protein antigens as reagents and vaccine candidates, with a significant reduction of production costs when compared to more traditional solutions; a recent study [12] estimated that approximately 9400 kg of plant biomass (12,500 sq. m of greenhouse space) would be sufficient to satisfy the demand of the virus-like particle vaccine against COVID-19 for an Italian population with only 10% of the capital costs required for fermenter-based infrastructure. The main limitations are related to public acceptance and to the management of protocols for plant cultivation, given that plant-based vaccines can be produced only through genetically modified plants. Several studies have analyzed the scenario of plant-based vaccines production against COVID-19 from a technical perspective [13,14,15,16] outlining the enormous potential of this technique to provide safe and affordable tools to fight the current pandemic, but also the challenges related to plant expression, as dosage consistency. Kumar et al. [17] presented an updated overview of the main features of existing plant-based vaccine candidates focusing on the technological challenges related to the development of these highly innovative products. Nevertheless, to the best of our knowledge, there is a lack of studies investigating the innovation scenario itself, to evaluate the perspectives for the development of these technologies and their potential role in contributing to develop solutions.

In this context, the aim of the present paper is to explore the innovation scenario of plant-based vaccine production by using the patent literature to identify the main innovation paths, the major and minor players who contribute to the production of plant-based vaccines, and to provide a worldwide perspective of the innovation trends for plant-based vaccines production.

Patent data has been selected as an innovation indicator as a large body of literature has assumed that the number of patents mirrors to a certain degree the changes in technological development. Since patents are normally published 18 months after their application, the present work is not able to fully trace the innovative activity connected with the outbreak of the COVID-19 pandemic. Nevertheless, the analysis of patent evolution in plant-based vaccines production will allow the description of the state of the art of research in this field, the identification of the know-how acquired, and consequently some forecasts about the possible developments in this sector of innovation in the near future.

## 2. Materials and Methods

The analysis of patents was carried out starting from an extraction on 2 January 2021 from Questel’s IP Business Intelligence application “Orbit Intelligence”, which allows the consultation of the English version of patent documents published worldwide. The search was based on two different query strategies composed of technical codes and keywords search [18], while technical codes represent a hierarchical classification system used primarily to classify patent documents according to the technical fields of inventions: IPC (International Patent Classification) codes have been established by the Strasbourg agreement in 1971 and represent the most used patent classification system worldwide [13], whereas the more recent Cooperative Patent Classification (CPC)—developed jointly by the European Patent Office (EPO) and the US Patent Office (USPTO)—has acquired relevance and international scope over the last few years and currently many international patent systems (including the Chinese patent system) are adopting this classification scheme [19,20].

The IPC/CPC query focused on a combination of three main technology fields—biotechnology, agriculture, and medicine—linked together to identify the specific innovation sector related to plant-based production of biopharmaceutical compounds. The first part of the string regarded the specific field of plant biotechnology (C12N 15/8257 OR C12N 15/8258 codes) and the inventions related to the modification of plant genome to produce pharmaceutical products and vaccines. The second part of the string aimed at identifying a more global group of inventions that involves agriculture (A01H 1 code) for the production of medical products (A61K and A61P codes). The selection of keywords included the terms “plant” and “vaccine”, plus an additional focus on the term “virus-like particles” (VLP), which is one of the most interesting strategies to develop innovative vaccines [21,22]. For each keyword selected, the use of the “+” symbol allowed us to make the research more inclusive and complete (i.e., Rotarivirus-like particles). Table 1 summarizes the specific codes used in the search phase and provides some additional details for each IPC/CPC code technical description.

The IPC/CPC-based extraction yielded 2041 patent families (14,838 patents), while the second keyword-based query resulted in 311 patent families (4493 patents).

The set of technical codes used (Table 2) were designed to identify the whole scenario of innovation related to biopharmaceutical-medical drugs produced using biotechnology production in plants.

**Figure 1:**
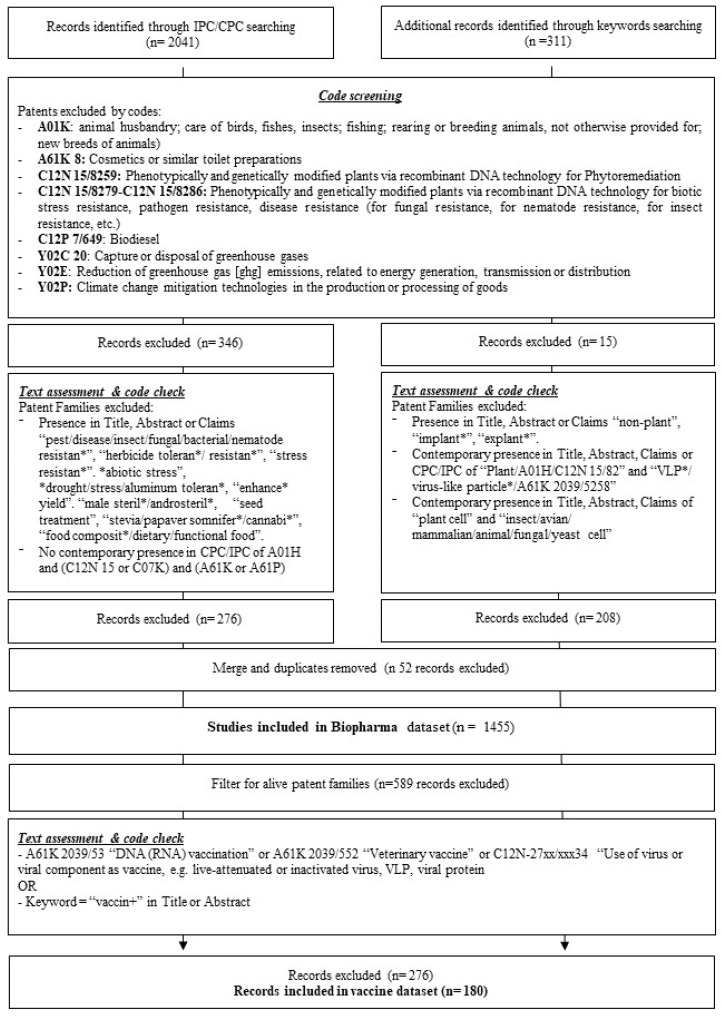
Flowchart for data cleaning operations.

A series of cleaning operations were performed to exclude all those records that did not fully match the scope of the selected innovation sector. More specifically, a list of IPC/CPC codes (Figure 1) that in general terms could be part of the search strategy output, but in fact refer to different applications and inventions (i.e., animal breeding, or cosmetics), were searched for in the dataset, and the corresponding patent families were excluded. Then, an additional text assessment and code check allowed the identification, and subsequent exclusion, of patent families regarding pest and disease control in plants (keywords “pest/insect/fungal/nematode resistance”, “herbicide tolerance”), and the remaining records were manually screened to eliminate spurious records. Data cleaning operations lead to a dataset composed of 1455 patent families (corresponding to 8234 patents) regarding the global scenario of biopharma inventions, which were successively filtered for the legal status “alive” to exclude expired or lapsed patent families. A further level of analysis was performed by applying to the original biopharma dataset some keyword-based filters that detected a more specific innovation context, the production of vaccines (Figure 1), leading to a final dataset composed of 180 patent families corresponding to 1397 single patents.

Data elaboration was performed making the distinction between patent family and patents. Patent family relates to a set of patents of the same invention, granted anywhere in the world (usually the first publication is in the assignee’s domestic patent office); statistics based on the count of patent families provide information on the origin of innovation itself, and the size of patent family (in terms of the number of patents) is considered a proxy for the value of the invention. Conversely, the term patent regards the single patent document, and this variable is more indicative of the spread of innovation and its target market.

The analysis was performed through descriptive statistics of raw patent data according to four different levels: time trends, country analysis, assignee classification, and object of the inventions.

Time trends, intended as the evolution over time of patent data, were evaluated as the number of patent families/years, considering both the priority year and the publication year. The first variable was defined as the filing date of the first application for a given invention and was shared by the whole patent family. Conversely, the variable publication year referred to a single patent document. Country analysis was performed through the elaboration of the following indices:

Total patent families by priority country, as the sum of the patent families sharing the same priority country. When the priority derives from an international procedure (under the Patent Cooperation Treaty—PCT), patent families were assigned based on the second country code reported in the family priority details;

- Average number of patents: a variable that provides information about the mean value for the size of patent families, intended as the number of patents that compose it;
- Average age: a country-based index that accounts for the mean number of years of each patent family (based on the priority year);
- Non-self forward citations: patent families of a single country according to the average number of (non-self) citations a patent receives and considered as a measure of the technological impact of inventions [23];

For the assignee analysis, the construction of the variables aimed to describe and characterize the type of players involved in this specific innovation sector. More specifically, assignee analysis was performed through the elaboration of the following variables:

- Assignee name: name of the current owner of the patent family. A subsequent analysis from the assignee’s point of view was first performed to track mergers and acquisitions and assign patent ownership according to the present corporate asset (i.e., Corteva includes Dow and DuPont patents);
- Assignee country: assignee’s country of residence;
- Typology of assignee: classification based on public/private nature of the patent’s owner. More specifically, public institutions were categorized as university, government, or non-profit. The private sector was generally classified as private, except for the identification of the four main agbiotech companies: Bayer (including Monsanto), Corteva (Dow and DuPont), Syngenta, and BASF;
- Number of patent families and number of patents: absolute values for the count of patent families and related patents owned by each assignee;
- Patents/family: index accounting for the average size of patent families, intended as the number of patents that compose them. The size of patent families is normally related to the value of inventions [24];
- Non-self forward citations: describes patent families of a single assignee according to the average number of (non-self) citations a patent receives and is considered as a measure of the technological impact of inventions [23];
- Non-self forward citations/family: index developed to outline the average number of (non-self) forward citation for each patent family;
- Average age of patent families: an assignee-based index that account for the mean number of years of each patent family;
- Percentage of patents > 2016: number of patents with application date prior to the year 2016, accounting for the innovation intensity in the most recent period;
- Percentage of patent families patented in US-EP: index developed to reveal the rate of innovation protection in main patent systems, which is conventionally considered an indicator of patent value [24].

As for the object of the invention, the analysis aimed at identifying the profile of technological classification for each patent family as described by the use of the International Patent Classification (IPC) and Cooperative Patent Classification (CPC) codes, classifying every single invention for function or by its field of application [25]. Since a patent may contain several technical objects and therefore can be assigned to several IPC/CPC classes, the relative frequency of technical codes may exceed 100%. The analysis regarded the CPC codes of three main classes:

- A61: Medical or Veterinary Science; Hygiene.
- C07: Organic Chemistry.
- C12: Biochemistry; Beer; Spirits; Wine; Vinegar; Microbiology; Enzymology; Mutation or Genetic Engineering.

Inside these three classes, ten main aggregates have been considered:

- Two sub-classes
  1. A61P Specific therapeutic activity of chemical compounds or medicinal preparations;
  2. C12P Fermentation or enzyme-using processes to synthesize a desired chemical compound or composition or to separate optical isomers from a racemic mixture.
- Five Groups
  3. A61K 38 Medicinal preparations containing peptides;
  4. A61K 39 Medicinal preparations containing antigens or antibodies;
  5. C12N 27 Viruses;
  6. C07K 14 Peptides having more than 20 amino-acids; gastrins; somatostatins; melanotropins; derivatives thereof;
  7. C07K 2319 Fusion polypeptide.
- One sub-group
  8. C12N 15/82
- Two Combinations of groups and/or subclasses
  9. C07K1 6 and C07K 2317 Immunoglobulins (Igs), e.g., monoclonal or polyclonal antibodies; immunoglobulins specific features.
  10. C12N 9 and C12Y Enzymes; proenzymes; compositions thereof.

Five of these ten aggregates (namely: 1; 4; 5; 6; 8) were further disaggregated in order to catch some specific sub-classifications of major importance in the study, as reported in Appendix A.

## 3. Results

### 3.1. The Role of Vaccines in the Biopharmaceutical Sector

**Figure 2:**
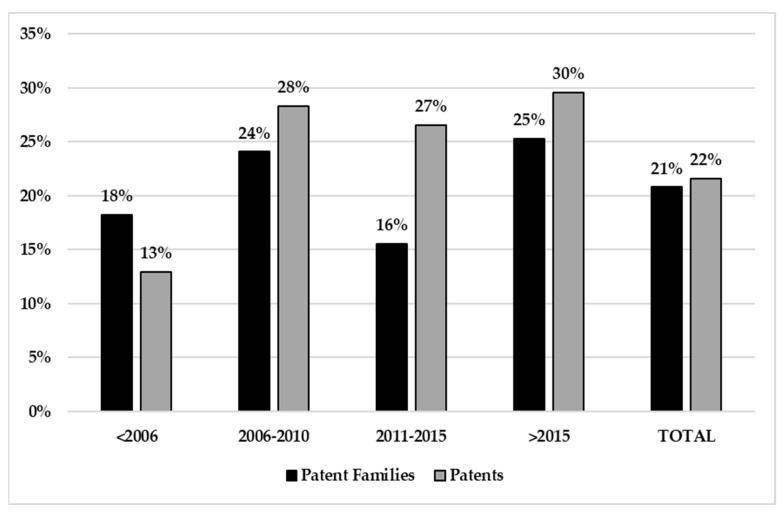
Patent statistics in biopharma vs. vaccine production.

The patent data show that innovation in biopharmaceutical production resulted in roughly two hundred new patent families in each five-year period considered, with quite a stable trend over time. It is not the same for the single patent count, which reveals a significant decrease over time, starting from 1950 in the 2006–2010 period to 568 in the last five-year period considered (Figure 2). Plant-based vaccines are part of this innovative sector covering on average 21% of patent families and 22% of patents, though with some fluctuation over years.

This section may be divided by subheadings. It should provide a concise and precise description of the experimental results, their interpretation, as well as the experimental conclusions that can be drawn.

### 3.2. Time Trends

**Figure 3:**
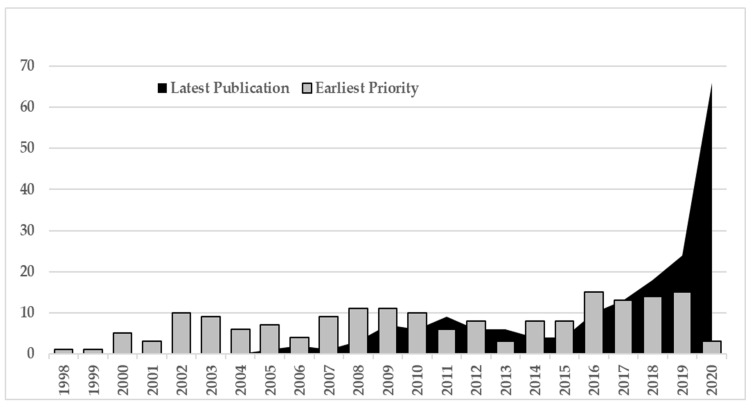
Evolution of plant-based vaccines patent application over time.

The evolution of patent applications over time for plant-based vaccines (Figure 2) can be deduced by first considering the variable first priority year, which is the year of the first request for protection of a given invention worldwide (Figure 3, grey bars). At the global level, the data shows that this set of technologies began to develop in late 1990, and that the number of patent applications remained quite constant until the year 2015. Since then, patent applications have constantly grown and almost doubled from the 2011–2015 period (33 patent families) to the 2016–2020 period (60 families), even though data from 2020 must be considered incomplete (due to the time-lag between patent application and publication).

This growing trend is more evident when considering the data related to the last publication year (Figure 3, dark area), which shows a dramatic increase in patent publications in the last five-year period.

Since the variable last publication year refers to the evolution of patent publication within each single patent family, which is composed of a set of patents referring to the same invention but protected in different patent systems, this data outlines that in recent years, there has also been a significant increase in the diffusion of innovation, in addition to the mere inventive activity revealed by the trend in the priority year. The dynamics of the patent diffusion are detailed in Table 3, where the data shows that, in general terms, patent families evolved over time following a quite established development dynamic; more specifically, results show that every patent family had, on average, two patent publications in the five-year period when the priority was requested, then in the subsequent five-year period, the number of patent publications reached their maximum peak, while in the third five-year period, the number of publications declined. Notably, the evolution over time shows that the peak reached a growing number of patents counted (3.8 before 2006, 5.6 between 2006 and 2010, and 8.1 for the 2011–2015 period), so that it is possible to predict that the patent families published in the last period will generate a number of patents higher than the previous periods.

In summary, the results of the time trend analysis reveal that plant-based vaccines represent an innovation sector that has acquired increasing importance in recent years, and consequently a boost in the development and commercialization of new plant-based vaccines can be assumed in the near future.

### 3.3. Country Analysis

**Figure 4:**
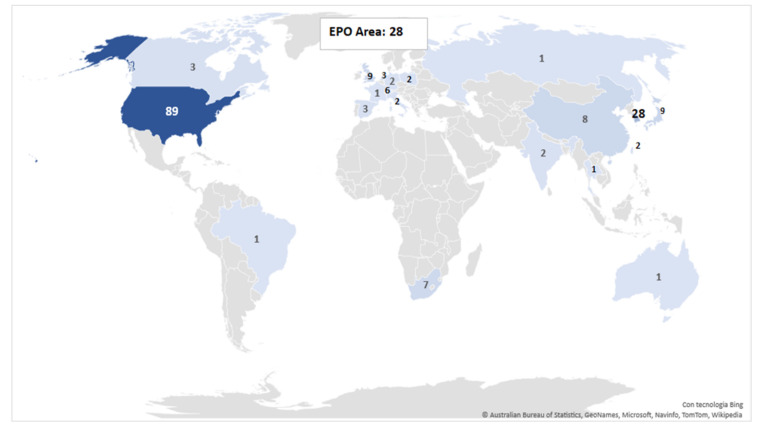
Distribution of patent families by first priority country.

**Figure 5:**
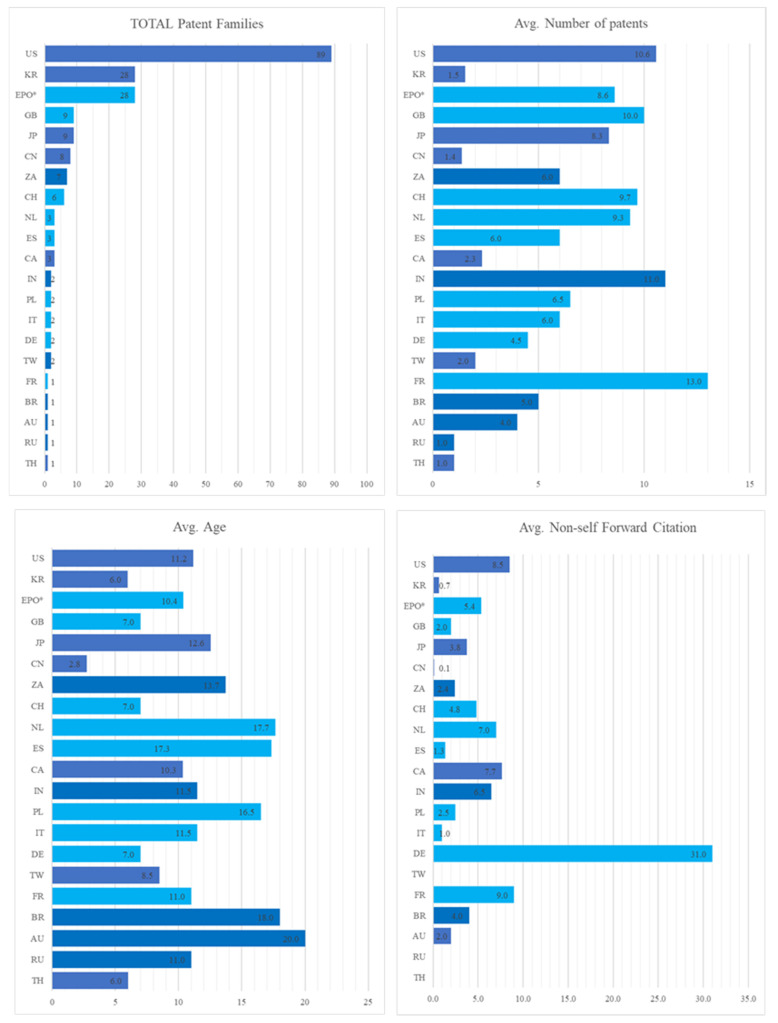
Country-based patent indices. Source: authors elaboration based on Questel-orbit data.

Country analysis based on the priority country reveals that the innovation activity in this field of research is not equally distributed worldwide. The United States is the leading country with 91 families, representing 50% of the total patenting production (Figure 4). Far below, the Rep. of Korea and the EPO area are represented both with 28 patent families: the member states which mostly contribute to the European area activity are Great Britain (nine families) and Switzerland (six families). A minor role is played by Japan and China (nine and eight patent families, respectively), while the remaining countries account for only 8% of the total. A more detailed description of the trends in country-based patent production is provided by Figure 5, where a set of four indices—total patent families, the average number of patents, average age, and average non-self forward citations—was developed. Results indicate that the United States is the leading patent system, not only for the number of patent families (meaning invention attractiveness), but also for the value of innovation, given that the average number of patents for each family is much higher than the Rep. of Korea and the EPO area (10.5 vs. 1.5 and 8.6, respectively).

In general, this latter index highlights huge differences in patenting strategy among countries: a group composed, inter alia, by the United States, Switzerland, Great Britain, and the Netherland have a clear propensity to protect inventions in a large number of patent systems worldwide (with index “average number of patents” higher than nine).

In contrast, other countries, and in particular eastern economies (China and the Rep. of Korea especially), though having a leading position for the number of patent families, show a quite minor attitude to disseminate their inventive activity; the index assumes that values below two indicate that inventions are often only protected in the domestic patent system.

As for the index “average age”, results show that among the countries that have started to develop innovation in plant-based vaccine production, some are not the current leaders in this technology; indeed, Australia and Brazil have the highest value for this index (20 and 18 years, respectively), followed by the Netherlands and Poland, whereas top countries like the US and the EPO area started to produce inventions in this field approximately ten years ago, with the exception being the Rep. of Korea where the research in this sector started some years later. As for the index “average non-self forward citation” the index reveals a quite uniform behavior, with all the countries ranging below the value of ten citations, except for Germany where a single patent family (EP262312, owned by the company Icon Genetics) received 62 non-self forward citations.

In summary, the country analysis shows the presence of one relevant innovation area, the United States, standing out as the most interesting market for plant-based vaccine production. Nevertheless, the future scenario will probably also include the European Union and some eastern economies like the Rep. of Korea.

### 3.4. Assignee Analysis

As for the analysis of the type of assignee, the results shows a quite balanced distribution among the different categories of players: globally, 49% of patent families belong to private assignees (or derive from private–private collaboration), 37% derive from public research activity (or public–public collaboration), while 12% of patent families come from public–private synergies (i.e., KR101732624 owned by the Korea Rural Development Administration and the Korean company Bioapplications or EP3167057 owned by Medicago together with the Canadian University of Laval), while the remaining 2% pertain to single inventors.

Table 4 outlines the results for the assignee classification based on their strategic positioning and productivity over time; more specifically, considering the variables average age of patent families, number of patent families, number of non-self forward citations, and number of patents after 2016, assignees have been classified into three main groups: pioneers, leaders, and newcomers. Pioneers are characterized by having the highest value for the variable average age of patent families and the lowest value for the variable number of patents after 2016; this means that this group of assignees worked in plant-based vaccine production in the earliest years of development of this innovation sector, though they do not occupy a dominant position in the current period. The group of assignees defined as leaders is characterized by a lower value for the average age of patent families (between 16 and 8 years) and higher levels of patent productivity after 2016 (greater than 10%). The last group of assignees is classified as newcomers because it is defined by the lowest value for average patent age (lower than 8 years) and the highest productivity in the last five-year period.

Table 4 outlines the results of patent-based indices elaboration for each of the assignee groups identified. The pioneers group of assignees is fully composed of US players, both public and private. Among the public institutions can be found one university (Ohio State, strongly focused on veterinary vaccines) and a non-profit institute (Boyce Thompson Institute for Plant Research). For the private sector, the most significant player is represented by the firm Kentucky Bioprocessing, the biotech subsidiary of the company British American Tobacco; this firm has announced the development of a COVID-19 vaccine candidate, which is currently in phase 1/2 of clinical testing. In 2014, the company produced an anti-Ebola monoclonal antibody cocktail, and more importantly, a vaccine platform technology developed for pandemic flu, which was then exploited as the core of their COVID-19 vaccine development. Moreover, amongst leaders, there is the presence of only one agbiotech firm (Corteva) of the so called “Big Six” (recently turned to be “Big Four” due to mergers and acquisitions), which have historically played a leading role in the research and development of agbiotech innovations. The remaining two players are Advanced Bionutrition, specializing in veterinary vaccines, and Pfenex (rebranded in 2021 to Pelican Expression Technology), which inherited part of the patents developed by DowAgroSciences.

The structure of the patenting activity of the leaders group of assignees (Table 5) confirms the US leadership already evidenced in the country analysis, given that among the nine top players identified, six originated in the United States. Nevertheless, the first assignee in terms of the number of patent families is the Canadian firm Medicago, with 21 families corresponding to the impressive amount of 466 single patents. This data can certainly explain the fact that Medicago was the first company to develop a COVID-19 plant-based vaccine candidate since the Canadian company had already developed the experience and the technologies for vaccine production, and subsequently had the opportunity to adapt existing solutions for the case of COVID-19. Medicago, since 2013, has been owned by a subsidiary of Mitsubishi Tanabe Pharma (67%) and Phillip Morris International (33%), and has been very active in the fields of antiviral vaccines and antibody therapeutics.

A further leader player is represented by a US public institute, the Arizona State University, with six patent families, has a considerably smaller portfolio in terms of the number of patents when compared to Medicago (19 patents), confirming a very common behavior of the public sector, which is the ability to innovate and produce inventions paired with the lack of attitude and capacity to exploit the inventions and disseminate them. Notably, a second company that has already announced the development of a COVID-19 vaccine candidate is on the list of the leader assignee: this is the US firm iBio, which is highly specialized in plant-based biologics manufacturing and owns four patent families composed of forty single patents. This company has already produced a plant-based animal vaccine against swine fever and is working on the development of a second-generation vaccine, which they have announced will be able to overcome some of the limitations inherent in the currently available products: cold chain, cost, and protection against variants.

A further point of interest regarding this group of players is related to the patenting strategy and can be recognized by the variable percent of families patented in US-EP, which show a clear attitude to protect inventions in major patent systems, even when they do not coincide with the domestic one, as the Spanish firm Zip Solutions (100% of patent families in the US) and the Canadian firm Medicago (20 patent families out of 21 patented in the US). The public institute Fraunhofer, though headquartered in Germany, has several research centers in the United States and has developed patents for a plant-based vaccine, and has been consequently been classified as US assignee. A further firm belonging to this group of assignees is the Spanish biotech company Zip Solutions, which specializes in recombinant vaccines for human and animal health and in research on virus-like particles.

The last group of assignees, classified as newcomers, is characterized by the largest variety of players, with an increased role of public institutions: six assignees belong to universities and public research centers, and also the Swiss firm Saiba AG, founded in 2012, is a spin-off of the University of Zurich. This company also announced that it is working on a future COVID-19 vaccine candidate, focusing on the implementation of a second-generation vaccine product be developed in the coming years. Saiba AG and the University of Zurich present high values for the number of patents (24 and 18, respectively) together with the highest values for patents/family, and consequently can be recognized as the most promising players among the newcomers group. Finally, Plantform, is a biotechnology company that produce vaccines and antibodies in tobacco plants using a technological platform developed at the University of Guelph.

Table 6 summarizes the main features of patent production for each type of assignee. In general terms, the nine players identified as leaders own 26.7% of the inventions; thus, it is possible to say that this specific innovation sector is characterized by a quite low concentration. Nevertheless, the concentration level significantly increases and reaches a value of 52.3% when considering single patent count. By contrast, the role of the group of assignees classified as newcomers shows an opposite trend, since their relevance significantly decreases from patents families (26.7%) to single patents count (10.9%).

**Figure 6:**
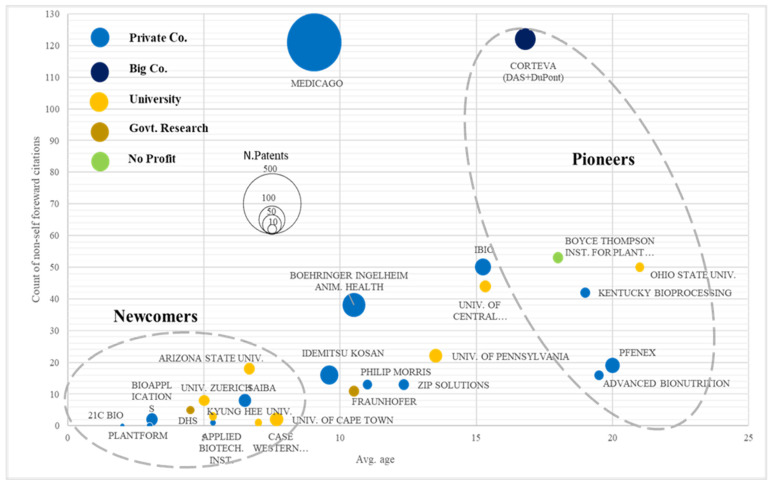
Assignee’s analysis–synthetic map.

Figure 6 condenses the different profiles of assignees according to three main variables: age, count of non-self forward citations, and number of patents. The map shows that the intense development of this innovative sector generated one leading player, the company Medicago, which detaches itself from the rest of the assignees for the number of patents and citations. Data suggest that this firm is the one characterized by the most intense research background and these results could partially explain the current position of Medicago having the most advanced COVID-19 plant-based vaccine candidates. In a similar position was the company Corteva, though its share of patents after 2016 decreased to 1.5%, meaning that after an intense phase of innovation production, in the last period, the company decided to invest in different fields of research. Of the COVID-19 plant-based vaccine candidates, one was developed by a pioneer assignee (Kentucky Bioprocessing), while two of them belong to leader assignees (Medicago and iBio); albeit results outline a clear leading position for the company Medicago, some similarities can be found between the profile of the firms Kentucky Bioprocessing and iBio for the variables considered (average age and count of non-self forward citations). The bottom-left area of the map is crowded by a huge number of new players who recently entered this innovation sector with potential future development in the coming years.

In brief, the assignee analysis revealed the dominance of the public sector and the presence of a high variety of players, led by a few, specialized companies focused more on the protection of plant-based inventions, and also are connected to COVID-19 vaccine candidate development.

### 3.5. Object of the Invention

**Figure 7:**
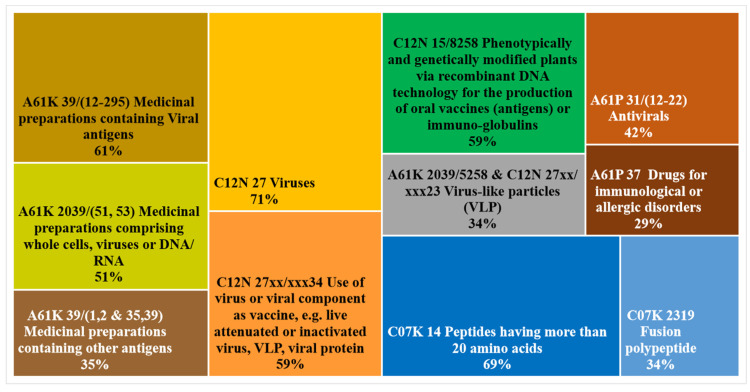
CPC codes distribution for plant-based vaccines.

The CPC codes analysis (Figure 7) reveals a quite consistent technical profile of inventions, which mainly fall in the domain of medicinal preparation (A61K codes) and plant biotechnology (C12N codes). It can be seen that the most used technical code is the C12N 27, which describes inventions related to viruses, while one-third of the inventions also contains the code for virus-like particles. Nevertheless, when analyzing technical code distribution, it is important to consider that the attribution of codes is highly dependent on the patent examiner and consequently highly variable. The time evolution of codes for plant-based vaccines (Appendix A and Appendix B) adds some relevant details about the pattern of CPC used in the considered period.

**Figure 8:**
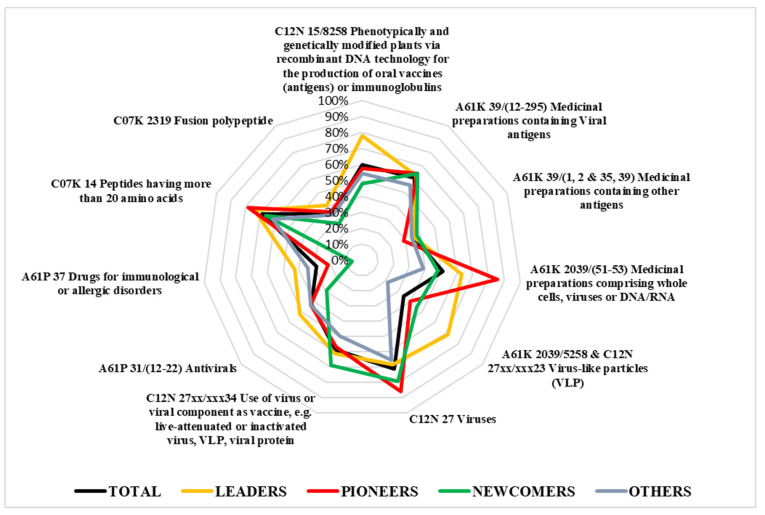
IPC/CPC distribution by typology of assignees.

Figure 8 provides the radar plot of the CPC distribution by typology of assignees. Results show that the process of innovation production—reflected by the selection of CPC codes—present some differences depending on the type of assignee considered; more specifically, leaders (yellow line) are characterized by intense use of the code C12N 15/8258 regarding the development of genetically modified plants for the production of vaccines. This aspect highlights the leading role of this group of players as they are highly specialized in applied research for the realization of new potential commercial products.

The second point of interest related to the leaders’ group is their expertise in the field of virus-like particles implementation (codes A61K 2039/5258 and C12N 27xx/xxx23), which is one of the most promising technologies for plant-based vaccine production. By contrast, pioneers firms (red line) have a tendency to be more specialized in medicinal preparations comprising whole cells, viruses, or DNA/RNA (codes A61K 2039/(51–53) and C12N 27).

**Figure 9:**
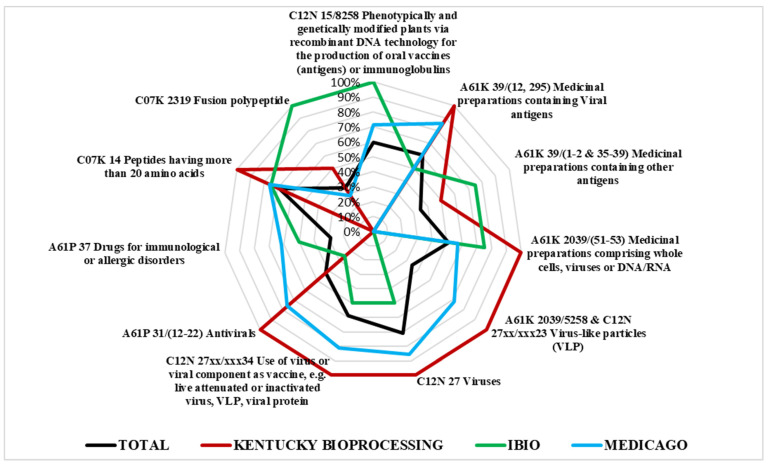
IPC/CPC distribution for the companies with a COVID-19 vaccine candidate.

A more detailed scenario is provided by Figure 9, where the general CPC distribution (black line) is compared to the ones of the three companies having announced their obtainment of a COVID-19 vaccine candidate. The company Medicago (blue line) shows a very balanced pattern of technological development, with the highest percentages in the key codes for virus-like particles. The company Kentucky Bioprocessing shows quite a different profile, which is more focused on virus research, while the codes for plant biotech applications are almost absent. Finally, the firm iBio is characterized by the intense use of the code C12N 15/8258, which is present in 100% of the patent families registered, whereas the research on virus-like particles does not seem to be the focus of this company. Nevertheless, its activity is not only dedicated to viruses, since two of the four patent families owned by this company focus on the treatment of Plasmodium and Trypanosoma diseases. Further information about CPC distribution by type of assignee is in Appendix B.

In summary, the analysis of CPC technical classification shows the emergence of virus-like particle technology for vaccine production, and a greater specialization in biotechnology applications for those firms who have already developed a COVID-19 vaccine candidate.

## 4. Conclusions

Plant-based vaccine development and production is a field of research and innovation characterized by positive evolution over time. Patent data analyzed refer to the pre-pandemic scenario, and yet the margins for the expansion of this sector are prominently displayed; most probably the event of the COVID-19 pandemic will act as an innovation catalyst so that it is possible to expect a dramatic intensification of the research activity in this innovation field.

Patent data analysis revealed that innovation in plant-based vaccines is mostly focused in the US for its capacity to attract domestic and foreign inventions, while the Rep. of Korea and the European area present a second, but much lower, position. Notably, results also indicate that the Chinese innovation sector, traditionally playing a leading role for research in biotech applications, is almost absent from the patent dataset at the moment. Moreover, assignee analysis shows that the private sector is dominant; in most cases, the research originated from small/medium firms or university spin-offs, then funded by big private companies, whereas by contrast, patent analysis shows the marginal role of public research in the leaders group of assignees. Results also confirm the central role of the research on virus-like particles, since two of the players who have already developed a vaccine candidate are the ones more specialized in this field. In addition, the present study also certifies the relationship between a firm’s patent portfolio and its innovation capacity, since the companies that had already developed plant-based COVID-19 vaccine candidates stand out as key-players in the patent analysis. Nevertheless, other common factors can contribute to explain this success, such as the ownership of plant-based production platforms (Medicago, iBio) and the presence of funding partners or technological collaboration (GlaxoSmithKline and Medicago) with pharmaceutical companies. In conclusion, a final keyword search among titles and abstracts of the patents contained in the dataset allowed the identification of one single patent related to the coronavirus (CN111254155, titled “Method for expressing viral vaccine by using plant as host” and owned by a Chinese inventor, published in 2020). Nevertheless, due to the patent document’s secrecy period, this result was highly expected as the first substantial wave of COVID-19-related inventions will be published from late 2021. Thus, the future perspectives of the present work are to understand how the COVID-19 pandemic boosted research in this field and eventually modified the trends in patenting activity, and to trace the development of new authorized plant-based vaccines through appropriate market indicators. Moreover, this work also confirms the power of patent data and their useful role in identifying the structure and the dynamics of very specific innovation sectors.

## Figures and Tables

**Table 1:** Plant-based vaccine candidates. Source: WHO.

Phase	Developers	Vaccine Platform Description	Type of Candidate Vaccine	Number of Doses	Schedule	Route of Administration
Phase 3	Medicago Inc.	Virus like particle	Coronavirus-Like Particle COVID-19 MT-2766	2	Day 0 + 21	Intra muscular
Phase 1/2	Kentucky Bioprocessing Inc.	Protein subunit	KBP-COVID-19 (RBD-based)	2	Day 0 + 21	Intra muscular
Phase 1	Baiya Phytopharm Co., Ltd.	Protein subunit	Baiya SARS-CoV-2 VAX1, a plant-based subunit vaccine (RBD-Fc + adjuvant)	2	Day 0 + 21	Intra muscular
Pre-Clinical	iBio/CC-Pharming	Protein subunit				
Pre-Clinical	Baiya Phytopharm/ Chula Vaccine Research Center	Plant-based subunit (RBD-Fc + Adjuvant)				
Pre-Clinical	Akdeniz University, Department of Agricultural Biotechnology, Antalya, Turkey	Development of recombinant protein based S1 and S2 (Spike) and nucleocapsid subunits vaccines using a plant expression vector.				
Pre-Clinical	Shiraz University	Plant derived VLP				

**Table 2:** Query strategy for patent collection.

(C12N 15/8257 OR C12N 15/8258)/CPC OR (((C12N 15 AND A01H 1) OR (C07K AND A01H 1)) AND (A61K OR A61P))/IPC/CPC	
C12N 15/8257	Phenotypically and genetically modified plants via recombinant DNA technology for the production of primary gene products, e.g., pharmaceutical products, interferon
**or**	**or**
C12N 15/8258	for the production of oral vaccines (antigens) or immunoglobulins
**OR**	**OR**
C12N 15	Mutation or genetic engineering/ DNA or RNA concerning genetic engineering, vectors, e.g., plasmids, or their isolation, preparation or purification/ Use of hosts therefor
**and**	**and**
A01H 1	New Plants or Processes for Obtaining Them/ Plant Reproduction by Tissue Culture Techniques: Processes for modifying genotypes
**OR**	**OR**
C07K	PEPTIDES
**and**	**and**
A01H 1	New Plants or Processes for Obtaining Them/Plant Reproduction by Tissue Culture Techniques: Processes for modifying genotypes
**AND**	**AND**
A61K	PREPARATIONS FOR MEDICAL, DENTAL, OR TOILET PURPOSES
**or**	**or**
A61P	SPECIFIC THERAPEUTIC ACTIVITY OF CHEMICAL COMPOUNDS OR MEDICINAL PREPARATIONS
**Keywords:**	plant+ AND vaccin+ AND (vlp+ OR (+virus like particle+) OR (+virus-like particle+))

**Table 3:** Patent distribution based on patent family publication period.

Earliest Priority Period	N. of Patent Families	Patents/Patent Family by Publication Period				
		<2006	2006–2010	2011–2015	>2015	Total
**<2006**	42	2.6	3.8	1.6	0.4	**8.4**
**2006–2010**	45	-	2	5.6	4.6	**12.3**
**2011–2015**	33	-	-	1.7	8.1	**9.8**
**>2015**	60	-	-	-	2.8	**2.8**
**TOTAL**	**180**	**0.6**	**1.4**	**2.1**	**3.7**	**7.8**

**Table 4:** Assignee classification.

	Pioneers	Leaders	Newcomers
**Av. Age of patent families (Years)**	>16	≤16 and ≥8	<8
**N. Patent Families Min**	>2	>2	>2
**N. non-self forward citations**	>10	>10	-
**Patents > 2016**	<5%	>10%	>50%

**Table 5:** Assignee description and patent statistics.

Assignee	Country	Typology		N. of Patent Families	N. of Patents	Patents/Family	Count of Non-Self Foreward Citations	Non-Self Foreward Citations/Family	Avg. Age of Patent Families	% Patents >2016	% Patent Families in US-EP
**“PIONEERS”**											
OHIO STATE UNIV.	US	PUB	UNIV	2	13	6.5	50	25.0	21.0	-	-
PFENEX	US	PRIV	PRIV	2	32	16.0	19	9.5	20.0	-	100.0
ADVANCED BIONUTRITION	US	PRIV	PRIV	2	13	6.5	16	8.0	19.5	-	50.0
KENTUCKY BIOPROCESSING	US	PRIV	PRIV	2	15	7.5	42	21.0	19.0	-	100.0
BOYCE THOMPSON INST.	US	PUB	NO PROFIT	2	17	8.5	53	26.5	18.0	-	50.0
CORTEVA (DAS+DuPont)	US	PRIV	BIG FIRM	5	66	13.2	122	24.4	16.8	1.5	80.0
**“LEADERS”**											
UNIV. OF CENTRAL FLORIDA	US	PUB	UNIV	3	20	6.7	44	14.7	15.3	10.0	33.3
IBIO	US	PRIV	PRIV	4	40	10.0	50	12.5	15.3	10.0	100.0
UNIV. OF PENNSYLVANIA	US	PUB	UNIV	4	26	6.5	22	5.5	13.5	38.5	75.0
ZIP SOLUTIONS	ES	PRIV	PRIV	3	17	5.7	13	4.3	12.3	47.1	66.7
PHILIP MORRIS	CH	PRIV	PRIV	2	14	7.0	13	6.5	11.0	14.3	100.0
BOEHRINGER INGELHEIM ANIM. HEALTH	US	PRIV	PRIV	4	80	20.0	38	9.5	10.5	67.5	100.0
FRAUNHOFER	US	PUB	GOV	2	17	8.5	11	5.5	10.5	64.7	100.0
IDEMITSU KOSAN	JP	PRIV	PRIV	5	51	10.2	16	3.2	9.6	62.7	100.0
MEDICAGO	CA	PRIV	PRIV	21	466	22.2	121	5.8	9.0	57.5	81.0
**“NEWCOMERS”**											
UNIV. OF CAPE TOWN	ZA	PUB	UNIV	6	27	4.5	2	0.3	7.7	59.3	50.0
CASE WESTERN RESERVE UNIV.	US	PUB	UNIV	2	9	4.5	1	0.5	7.0	88.9	-
ARIZONA STATE UNIV.	US	PUB	UNIV	6	19	3.2	18	3.0	6.7	68.4	33.3
SAIBA AG	CH	PRIV	PRIV	2	24	12.0	8	4.0	6.5	100.0	50.0
KYUNG HEE UNIV.	KR	PUB	UNIV	9	10	1.1	3	0.3	5.3	80.0	-
APPLIED BIOTECHNOLOGY INSTITUTE	US	PRIV	PRIV	3	5	1.7	1	0.3	5.3	80.0	-
UNIV. ZUERICH	CH	PUB	UNIV	2	18	9.0	8	4.0	5.0	100.0	100.0
DHS—SCIENCE & TECHNOLOGY DIRECT.	US	PUB	GOV	2	10	5.0	5	2.5	4.5	100.0	50.0
BIOAPPLICATIONS	KR	PRIV	PRIV	11	21	1.9	2	0.2	3.1	100.0	-
PLANTFORM	CA	PRIV	PRIV	2	6	3.0	0	0.0	3.0	100.0	50.0
21C BIO	FR	PRIV	PRIV	3	3	1.0	0	0.0	2.0	100.0	-

**Table 6:** Summary of the patent-based indices by assignee’s category.

Assignee	N. of Patent Families	N. of Patents	Patents/Family	Count of Non-Self Foreward Citations	Non-Self Foreward Citations/Family	Avg. Age of Patent Families	% Patents >2016	% Patent Families in US-EP
**“PIONEERS”**	15	156	10.4	302	20.1	18.6	0.6	66.7
**“LEADERS”**	48	731	15.2	328	6.8	10.9	53.5	83.3
**“NEWCOMERS”**	48	152	3.2	48	1.0	5.0	86.2	20.8
**“OTHERS”**	69	358	5.2	342	5.0	11.1	38.3	37.7
**GRAND TOTAL**	**180**	**1397**	**7.8**	**1020**	**5.7**	**10.1**	**47.2**	**47.8**
**“PIONEERS”/GRAND TOTAL**	8.3%	11.2%	1.3	29.6%	3.6	1.8	0.0	1.4
**“LEADERS”/GRAND TOTAL**	26.7%	52.3%	2.0	32.2%	1.2	1.1	1.1	1.7
**“NEWCOMERS”/GRAND TOTAL**	26.7%	10.9%	0.4	4.7%	0.2	0.5	1.8	0.4
**“OTHERS”/GRAND TOTAL**	38.3%	25.6%	0.7	33.5%	0.9	1.1	0.8	0.8

## Data Availability

Patent data are public and available on free access platforms (i.e., Espacenet, Google Patents). The data presented in this study are available on request from the corresponding author.

## Appendix A

**Table A1:** CPC Codes Distribution by Period.

CPC Code				CPC Description	TOTAL	<2006	2006-10	2011-15	>2015
**C12N 15/82**				**MUTATION OR GENETIC ENGINEERING; DNA OR RNA CONCERNING GENETIC ENGINEERING, VECTORS FOR PLANT CELLS**	**76.1%**	**76.2%**	**93.3%**	**69.7%**	**66.7%**
*Of Which:*	C12N 15/82(1-14)			Methods for introducing genetic material into plant cells,	15.6%	21.4%	13.3%	18.2%	11.7%
	C12N 15/82(16-39)			Methods for controlling, regulating or enhancing expression of transgenes in plant cells	7.8%	0.0%	17.8%	9.1%	5.0%
	C12N 15/82(41-98)			Phenotypically and genetically modified plants via recombinant DNA technology	73.9%	76.2%	88.9%	69.7%	63.3%
	*O/W:*	C12N 15/82(57-58)		for the production of primary gene products, e.g., pharmaceutical products, interferon	73.3%	76.2%	88.9%	69.7%	61.7%
		*O/W:*	*C12N 15/8258*	*for the production of oral vaccines (antigens) or immunoglobulins*	*59.4%*	*59.5%*	*73.3%*	*60.6%*	*48.3%*
**A61K39**				**MEDICINAL PREPARATIONS CONTAINING ANTIGENS OR ANTIBODIES**	**83.9%**	**88.1%**	**73.3%**	**90.9%**	**85.0%**
*Of Which:*	A61K 39/(0001-385)			Medicinal preparations containing antigens	78.3%	69.0%	71.1%	90.9%	83.3%
	*O/W:*	A61K 39/(12-295)		Viral antigens	61.1%	50.0%	53.3%	72.7%	68.3%
		*O/W:*	A61K 39/145	Orthomyxoviridae, e.g., influenza virus	15.0%	4.8%	31.1%	18.2%	8.3%
			A61K 39/215	Coronaviridae, e.g., avian infectious bronchitis virus	1.7%	4.8%	0.0%	0.0%	1.7%
		A61K 39/(1-2 & 35-39)		Other than viral (Bacterial, Fungal, Protozoa, etc.)	35.0%	47.6%	28.9%	36.4%	30.0%
	A61K 39/(395-44) & A61K 2039/505			Medicinal preparations containing antibodies; Immunoglobulins; Immune serum	1.7%	2.4%	0.0%	6.1%	0.0%
	A61K 2039/(51-53)			Medicinal preparations comprising whole cells, viruses or DNA/RNA	51.1%	59.5%	55.6%	54.5%	40.0%
	*O/W:*	A61K 2039/517		Plant cells	20.6%	31.0%	37.8%	12.1%	5.0%
		A61K 2039/525		Virus	31.1%	28.6%	24.4%	42.4%	31.7%
		*O/W:*	A61K 2039/5258	Virus-like particles	26.7%	21.4%	22.2%	36.4%	28.3%
		***A61K 2039/53***		***DNA (RNA) vaccination***	***5.6%***	***2.4%***	***8.9%***	***3.0%***	***6.7%***
	***A61K 2039/552***			***Veterinary vaccine***	***14.4%***	***14.3%***	***11.1%***	***18.2%***	***15.0%***
**A61K 38**				**Medicinal preparations containing peptides**	**6.7%**	**7.1%**	**2.2%**	**6.1%**	**10.0%**
**A61P**				**SPECIFIC THERAPEUTIC ACTIVITY OF CHEMICAL COMPOUNDS OR MEDICINAL PREPARATIONS**	**60.0%**	**71.4%**	**64.4%**	**48.5%**	**55.0%**
*Of Which:*	A61P 31			Antiinfectives, i.e., antibiotics, antiseptics, chemotherapeutics	47.8%	50.0%	53.3%	39.4%	46.7%
	*O/W:*	A61P 31/(12-22)		Antivirals	42.2%	47.6%	44.4%	33.3%	41.7%
		*O/W:*	A61P 31/(14-18)	for RNA viruses	32.2%	28.6%	35.6%	30.3%	33.3%
	A61P 37			Drugs for immunological or allergic disorders	28.9%	38.1%	44.4%	27.3%	11.7%
**C12N 27**				**VIRUSES**	**71.1%**	**66.7%**	**53.3%**	**81.8%**	**81.7%**
*Of Which:*	C12N 2760			ssRNA Viruses negative-sense	25.0%	16.7%	35.6%	24.2%	23.3%
	*O/W:*	C12N 2760/16		Orthomyxoviridae: Influenzaviruses	21.1%	7.1%	33.3%	21.2%	21.7%
	C12N 2770			ssRNA Viruses positive-sense	28.3%	35.7%	13.3%	39.4%	28.3%
	*O/W:*	C12N 2770/20		Coronaviridae	5.0%	4.8%	4.4%	0.0%	8.3%
	C12N 27xx/xxx23			Virus like particles [VLP]	27.8%	16.7%	20.0%	42.4%	33.3%
	*C12N 27xx/xxx34*			*Use of virus or viral component as vaccine,* e.g., *live-attenuated or inactivated virus, VLP, viral protein*	*58.9%*	*40.5%*	*48.9%*	*69.7%*	*73.3%*
**C07K 14**				**PEPTIDES HAVING MORE THAN 20 AMINO ACIDS; GASTRINS; SOMATOSTATINS; MELANOTROPINS; DERIVATIVES THEREOF**	**68.9%**	**69.0%**	**62.2%**	**69.7%**	**73.3%**
*Of Which:*	C07K 14/(5-19)			from viruses	50.6%	45.2%	37.8%	57.6%	60.0%
	C07K 14/(195-365)			from bacteria	11.1%	14.3%	11.1%	18.2%	5.0%
	C07K 14/(415-43)			from plants	5.0%	2.4%	13.3%	6.1%	0.0%
	C07K 14/(435-4748)			from animals	11.1%	9.5%	13.3%	3.0%	15.0%
**C07K 16 & C07K 2317**				**IMMUNOGLOBULINS [IGS], E.G. MONOCLONAL OR POLYCLONAL ANTIBODIES; IMMUNOGLOBULINS SPECIFIC FEATURES**	**11.1%**	**4.8%**	**6.7%**	**15.2%**	**16.7%**
**C07K 2319**				**FUSION POLYPEPTIDE**	**34.4%**	**40.5%**	**37.8%**	**33.3%**	**28.3%**
**C12N 9 & C12Y**				**ENZYMES; PROENZYMES; COMPOSITIONS THEREOF**	**8.9%**	**4.8%**	**17.8%**	**3.0%**	**8.3%**
**C12P**				**FERMENTATION OR ENZYME-USING PROCESSES TO SYNTHESISE A DESIRED CHEMICAL COMPOUND OR COMPOSITION OR TO SEPARATE OPTICAL ISOMERS FROM A RACEMIC MIXTURE**	**7.8%**	**4.8%**	**4.4%**	**9.1%**	**11.7%**
**TOTAL “VACCINE” PATENT FAMILIES (Absolute Values)**					**180**	**42**	**45**	**33**	**60**
***VLP TOTAL* ^[1]^**					**34.4%**	**21.4%**	**24.4%**	**48.5%**	**43.3%**

^[1]^ A61K 2039/5258 *OR* C12N 27xx/xxx23.

## Appendix B

**Table A2:** CPC Codes Distribution by Players—Pioneers and Leaders.

CPC Code				PIONE-ERS	OHIO STATE UNIV.	PFENEX	ADVAN-CED BIO-NUTRITION	KENTU-CKY BIO-PROCES-SING	BOYCE THOMP-SON INST. FOR PLANT RES.	CORTE-VA (DAS+Du Pont)	LEAD-ERS	UNIV. OF CEN-TRAL FLORI-DA	IBIO	UNIV. OF PENN-SYLVA-NIA	ZIP SOLU-TIONS	PHILIP MORRIS	BOEHR-INGER INGEL-HEIM ANIM. HEALTH	FRAUN-HOFER	IDE-MITSU KOSAN	MEDI-CAGO
**C12N 15/82**				**78.6%**	**100.0%**	**50.0%**	**100.0%**	**0.0%**	**100.0%**	**100.0%**	**95.8%**	**100.0%**	**100.0%**	**100.0%**	**100.0%**	**100.0%**	**75.0%**	**100.0%**	**80.0%**	**100.0%**
*Of Which:*	C12N 15/82(1-14)			21.4%	100.0%	50.0%	0.0%	0.0%	0.0%	0.0%	22.9%	66.7%	25.0%	100.0%	0.0%	50.0%	0.0%	50.0%	0.0%	9.5%
	C12N 15/82(16-39)			0.0%	0.0%	0.0%	0.0%	0.0%	0.0%	0.0%	20.8%	0.0%	25.0%	25.0%	100.0%	50.0%	0.0%	0.0%	0.0%	19.0%
	C12N 15/82(41-98)			78.6%	100.0%	50.0%	100.0%	0.0%	100.0%	100.0%	93.8%	100.0%	100.0%	100.0%	100.0%	100.0%	75.0%	100.0%	80.0%	95.2%
	*O/W:*	C12N 15/82(57-58)		78.6%	100.0%	50.0%	100.0%	0.0%	100.0%	100.0%	93.8%	100.0%	100.0%	100.0%	100.0%	100.0%	75.0%	100.0%	80.0%	95.2%
		*O/W:*	***C12N 15/8258***	***57.1%***	***0.0%***	***0.0%***	***100.0%***	***0.0%***	***100.0%***	***100.0%***	***79.2%***	***100.0%***	***100.0%***	***100.0%***	***33.3%***	***100.0%***	***75.0%***	***100.0%***	***80.0%***	***71.4%***
**A61K39**				**85.7%**	**100.0%**	**100.0%**	**100.0%**	**100.0%**	**50.0%**	**75.0%**	**85.4%**	**100.0%**	**100.0%**	**100.0%**	**33.3%**	**0.0%**	**100.0%**	**100.0%**	**100.0%**	**85.7%**
*Of Which:*	A61K 39/(0001-385)			64.3%	100.0%	50.0%	100.0%	100.0%	0.0%	50.0%	83.3%	100.0%	75.0%	100.0%	33.3%	0.0%	100.0%	100.0%	100.0%	85.7%
	*O/W:*	A61K 39/(12-295)		64.3%	100.0%	50.0%	100.0%	100.0%	0.0%	50.0%	64.6%	33.3%	50.0%	50.0%	33.3%	0.0%	100.0%	100.0%	20.0%	85.7%
		*O/W:*	A61K 39/145	14.3%	0.0%	0.0%	0.0%	0.0%	0.0%	50.0%	25.0%	0.0%	50.0%	0.0%	0.0%	0.0%	25.0%	50.0%	0.0%	38.1%
			A61K 39/215	0.0%	0.0%	0.0%	0.0%	0.0%	0.0%	0.0%	0.0%	0.0%	0.0%	0.0%	0.0%	0.0%	0.0%	0.0%	0.0%	0.0%
		A61K 39/(1-2 & 35-39)		28.6%	100.0%	0.0%	0.0%	50.0%	0.0%	25.0%	35.4%	100.0%	75.0%	75.0%	33.3%	0.0%	25.0%	50.0%	100.0%	0.0%
	A61K 39/(395-44) & A61K 2039/505			0.0%	0.0%	0.0%	0.0%	0.0%	0.0%	0.0%	0.0%	0.0%	0.0%	0.0%	0.0%	0.0%	0.0%	0.0%	0.0%	0.0%
	A61K 2039/(51-53)			85.7%	100.0%	100.0%	100.0%	100.0%	50.0%	75.0%	62.5%	100.0%	75.0%	75.0%	33.3%	0.0%	100.0%	100.0%	40.0%	57.1%
	*O/W:*	A61K 2039/517		35.7%	100.0%	0.0%	50.0%	0.0%	0.0%	50.0%	37.5%	100.0%	75.0%	75.0%	0.0%	0.0%	75.0%	0.0%	40.0%	19.0%
		A61K 2039/525		50.0%	0.0%	100.0%	50.0%	100.0%	50.0%	25.0%	35.4%	0.0%	0.0%	0.0%	33.3%	0.0%	50.0%	100.0%	0.0%	57.1%
		*O/W:*	A61K 2039/5258	35.7%	0.0%	50.0%	0.0%	100.0%	50.0%	25.0%	33.3%	0.0%	0.0%	0.0%	33.3%	0.0%	25.0%	100.0%	0.0%	57.1%
		***A61K 2039/53***		***0.0%***	***0.0%***	***0.0%***	***0.0%***	***0.0%***	***0.0%***	***0.0%***	***2.1%***	***0.0%***	***0.0%***	***0.0%***	***33.3%***	***0.0%***	***0.0%***	***0.0%***	***0.0%***	***0.0%***
	***A61K 2039/552***			***28.6%***	***100.0%***	***0.0%***	***100.0%***	***0.0%***	***0.0%***	***0.0%***	***16.7%***	***0.0%***	***25.0%***	***0.0%***	***0.0%***	***0.0%***	***100.0%***	***0.0%***	***60.0%***	***0.0%***
**A61K 38**				**21.4%**	**0.0%**	**50.0%**	**100.0%**	**0.0%**	**0.0%**	**0.0%**	**2.1%**	**0.0%**	**0.0%**	**0.0%**	**0.0%**	**0.0%**	**0.0%**	**0.0%**	**20.0%**	**0.0%**
**A61P**				**50.0%**	**0.0%**	**100.0%**	**0.0%**	**100.0%**	**50.0%**	**50.0%**	**75.0%**	**100.0%**	**75.0%**	**75.0%**	**100.0%**	**0.0%**	**100.0%**	**50.0%**	**60.0%**	**76.2%**
*Of Which:*	A61P 31			50.0%	0.0%	100.0%	0.0%	100.0%	50.0%	50.0%	64.6%	33.3%	50.0%	75.0%	33.3%	0.0%	100.0%	50.0%	60.0%	76.2%
	*O/W:*	A61P 31/(12-22)		**42.9%**	**0.0%**	**100.0%**	**0.0%**	**100.0%**	**50.0%**	**25.0%**	**54.2%**	**33.3%**	**25.0%**	**50.0%**	**33.3%**	**0.0%**	**100.0%**	**50.0%**	**0.0%**	**76.2%**
		*O/W:*	A61P 31/(14-18)	14.3%	0.0%	0.0%	0.0%	0.0%	50.0%	25.0%	52.1%	33.3%	25.0%	50.0%	33.3%	0.0%	75.0%	50.0%	0.0%	76.2%
	A61P 37			21.4%	0.0%	0.0%	0.0%	0.0%	50.0%	50.0%	45.8%	0.0%	50.0%	25.0%	100.0%	0.0%	25.0%	50.0%	20.0%	61.9%
**C12N 27**				**85.7%**	**100.0%**	**100.0%**	**100.0%**	**100.0%**	**50.0%**	**75.0%**	**66.7%**	**33.3%**	**50.0%**	**50.0%**	**33.3%**	**0.0%**	**100.0%**	**100.0%**	**40.0%**	**85.7%**
*Of Which:*	C12N 2760			35.7%	0.0%	50.0%	0.0%	100.0%	0.0%	50.0%	33.3%	0.0%	50.0%	0.0%	0.0%	0.0%	50.0%	100.0%	0.0%	47.6%
	*O/W:*	C12N 2760/16		21.4%	0.0%	0.0%	0.0%	50.0%	0.0%	50.0%	29.2%	0.0%	50.0%	0.0%	0.0%	0.0%	25.0%	100.0%	0.0%	42.9%
	C12N 2770			50.0%	0.0%	100.0%	50.0%	100.0%	50.0%	25.0%	25.0%	33.3%	0.0%	50.0%	0.0%	0.0%	25.0%	100.0%	20.0%	23.8%
	*O/W:*	C12N 2770/20		0.0%	0.0%	0.0%	0.0%	0.0%	0.0%	0.0%	2.1%	0.0%	0.0%	0.0%	0.0%	0.0%	0.0%	0.0%	0.0%	4.8%
	C12N 27xx/xxx23			28.6%	0.0%	50.0%	0.0%	100.0%	0.0%	25.0%	31.3%	0.0%	0.0%	0.0%	0.0%	0.0%	0.0%	100.0%	0.0%	61.9%
	***C12N 27xx/xxx34***			***57.1%***	***100.0%***	***50.0%***	***50.0%***	***100.0%***	***0.0%***	***50.0%***	***62.5%***	***33.3%***	***50.0%***	***50.0%***	***33.3%***	***0.0%***	***100.0%***	***100.0%***	***20.0%***	***81.0%***
**C07K 14**				**78.6%**	**100.0%**	**100.0%**	**50.0%**	**100.0%**	**50.0%**	**75.0%**	**79.2%**	**66.7%**	**75.0%**	**100.0%**	**100.0%**	**0.0%**	**100.0%**	**100.0%**	**80.0%**	**76.2%**
*Of Which:*	C07K 14/(5-19)			64.3%	0.0%	100.0%	50.0%	100.0%	50.0%	75.0%	54.2%	0.0%	50.0%	0.0%	33.3%	0.0%	100.0%	100.0%	40.0%	71.4%
	C07K 14/(195-365)			14.3%	100.0%	0.0%	0.0%	0.0%	0.0%	0.0%	18.8%	0.0%	25.0%	75.0%	0.0%	0.0%	25.0%	0.0%	80.0%	0.0%
	C07K 14/(415-43)			0.0%	0.0%	0.0%	0.0%	0.0%	0.0%	0.0%	8.3%	0.0%	0.0%	0.0%	100.0%	0.0%	0.0%	0.0%	0.0%	4.8%
	C07K 14/(435-4748)			7.1%	0.0%	0.0%	0.0%	50.0%	0.0%	0.0%	8.3%	33.3%	25.0%	25.0%	0.0%	0.0%	0.0%	50.0%	0.0%	0.0%
**C07K 16 & C07K 2317**				**7.1%**	**0.0%**	**0.0%**	**50.0%**	**0.0%**	**0.0%**	**0.0%**	**14.6%**	**0.0%**	**0.0%**	**0.0%**	**0.0%**	**0.0%**	**0.0%**	**0.0%**	**0.0%**	**33.3%**
**C07K 2319**				**35.7%**	**0.0%**	**100.0%**	**0.0%**	**50.0%**	**0.0%**	**50.0%**	**43.8%**	**33.3%**	**100.0%**	**75.0%**	**33.3%**	**0.0%**	**25.0%**	**100.0%**	**60.0%**	**28.6%**
**C12N 9 & C12Y**				**0.0%**	**0.0%**	**0.0%**	**0.0%**	**0.0%**	**0.0%**	**0.0%**	**20.8%**	**0.0%**	**75.0%**	**25.0%**	**0.0%**	**100.0%**	**0.0%**	**0.0%**	**0.0%**	**19.0%**
**C12P**				**14.3%**	**50.0%**	**50.0%**	**0.0%**	**0.0%**	**0.0%**	**0.0%**	**12.5%**	**0.0%**	**0.0%**	**0.0%**	**0.0%**	**50.0%**	**0.0%**	**50.0%**	**20.0%**	**14.3%**
**TOTAL “VACCINE” PATENT FAMILIES (Absolute Values)**				**14**	**2**	**2**	**2**	**2**	**2**	**4**	**48**	**3**	**4**	**4**	**3**	**2**	**4**	**2**	**5**	**21**
***VLP TOTAL* ^[1]^**				***40.0%***	***0.0%***	***50.0%***	***0.0%***	***100.0%***	***50.0%***	***25.0%***	***71.4%***	***0.0%***	***0.0%***	***0.0%***	***33.3%***	***0.0%***	***25.0%***	***100.0%***	***0.0%***	***71.4%***

^[1]^ A61K 2039/5258 *OR* C12N 27xx/xxx23.

**Table A3:** CPC Codes Distribution by Players—Newcomers and Others.

CPC Code				NEW-COMERS	UNIV. OF CAPE TOWN	CASE WEST-ERN RES. UNIV.	ARIZO-NA STATE UNIV.	SAIBA	KYUNG HEE UNIV.	AP-PLIED BIO-TECHNO-LOGY INSTI-TUTE	UNIV. ZUERICH	DHS-SCIENCE & TECHNO-LOGY DIRECT.	BIOAP-PLICA-TIONS	PLANT-FORM	21C BIO	OTH-ERS	TOTAL
**C12N 15/82**				**58.3%**	**100.0%**	**0.0%**	**83.3%**	**0.0%**	**11.1%**	**100.0%**	**0.0%**	**0.0%**	**100.0%**	**100.0%**	**0.0%**	**74.3%**	**76.1%**
*Of Which:*	C12N 15/82(1-14)			10.4%	33.3%	0.0%	33.3%	0.0%	0.0%	0.0%	0.0%	0.0%	9.1%	0.0%	0.0%	12.9%	15.6%
	C12N 15/82(16-39)			4.2%	0.0%	0.0%	0.0%	0.0%	0.0%	33.3%	0.0%	0.0%	0.0%	50.0%	0.0%	2.9%	7.8%
	C12N 15/82(41-98)			56.3%	100.0%	0.0%	83.3%	0.0%	11.1%	100.0%	0.0%	0.0%	90.9%	100.0%	0.0%	71.4%	73.9%
	*O/W:*	C12N 15/82(57-58)		56.3%	100.0%	0.0%	83.3%	0.0%	11.1%	100.0%	0.0%	0.0%	90.9%	100.0%	0.0%	70.0%	73.3%
		*O/W:*	***C12N 15/8258***	***47.9%***	***100.0%***	***0.0%***	***66.7%***	***0.0%***	***11.1%***	***100.0%***	***0.0%***	***0.0%***	***72.7%***	***50.0%***	***0.0%***	***54.3%***	***59.4%***
**A61K39**				**83.3%**	**100.0%**	**100.0%**	**83.3%**	**100.0%**	**77.8%**	**100.0%**	**100.0%**	**100.0%**	**72.7%**	**0.0%**	**100.0%**	**82.9%**	**83.9%**
*Of Which:*	A61K 39/(0001-385)			83.3%	100.0%	100.0%	83.3%	100.0%	77.8%	100.0%	100.0%	100.0%	72.7%	0.0%	100.0%	74.3%	78.3%
	*O/W:*	A61K 39/(12-295)		64.6%	100.0%	50.0%	50.0%	100.0%	55.6%	66.7%	0.0%	100.0%	63.6%	0.0%	100.0%	55.7%	61.1%
		*O/W:*	A61K 39/145	12.5%	0.0%	0.0%	0.0%	50.0%	44.4%	0.0%	0.0%	0.0%	9.1%	0.0%	0.0%	10.0%	15.0%
			A61K 39/215	2.1%	0.0%	0.0%	0.0%	0.0%	0.0%	0.0%	0.0%	0.0%	9.1%	0.0%	0.0%	2.9%	1.7%
		A61K 39/(1-2 & 35-39)		37.5%	0.0%	50.0%	33.3%	50.0%	66.7%	66.7%	100.0%	0.0%	9.1%	0.0%	100.0%	34.3%	35.0%
	A61K 39/(395-44) & A61K 2039/505			2.1%	0.0%	0.0%	0.0%	50.0%	0.0%	0.0%	0.0%	0.0%	0.0%	0.0%	0.0%	2.9%	1.7%
	A61K 2039/(51-53)			47.9%	66.7%	100.0%	50.0%	50.0%	11.1%	100.0%	100.0%	100.0%	18.2%	0.0%	100.0%	38.6%	51.1%
	*O/W:*	A61K 2039/517		10.4%	16.7%	0.0%	0.0%	0.0%	0.0%	100.0%	0.0%	0.0%	9.1%	0.0%	0.0%	12.9%	20.6%
		A61K 2039/525		35.4%	50.0%	100.0%	50.0%	50.0%	11.1%	0.0%	100.0%	100.0%	9.1%	0.0%	66.7%	21.4%	31.1%
		*O/W:*	A61K 2039/5258	33.3%	50.0%	100.0%	50.0%	50.0%	11.1%	0.0%	100.0%	100.0%	9.1%	0.0%	33.3%	15.7%	26.7%
		***A61K 2039/53***		***8.3%***	***16.7%***	***0.0%***	***33.3%***	***0.0%***	***0.0%***	***0.0%***	***0.0%***	***0.0%***	***0.0%***	***0.0%***	***33.3%***	***7.1%***	***5.6%***
	***A61K 2039/552***			***12.5%***	***0.0%***	***0.0%***	***16.7%***	***0.0%***	***0.0%***	***0.0%***	***100.0%***	***0.0%***	***18.2%***	***0.0%***	***33.3%***	***11.4%***	***14.4%***
**A61K 38**				**8.3%**	**0.0%**	**0.0%**	**0.0%**	**0.0%**	**0.0%**	**0.0%**	**0.0%**	**50.0%**	**9.1%**	**0.0%**	**66.7%**	**5.7%**	**6.7%**
**A61P**				**43.8%**	**16.7%**	**50.0%**	**50.0%**	**50.0%**	**11.1%**	**66.7%**	**100.0%**	**100.0%**	**45.5%**	**0.0%**	**100.0%**	**62.9%**	**60.0%**
*Of Which:*	A61P 31			35.4%	16.7%	0.0%	50.0%	50.0%	0.0%	66.7%	0.0%	100.0%	45.5%	0.0%	100.0%	44.3%	47.8%
	*O/W:*	A61P 31/(12-22)		**29.2%**	**16.7%**	**0.0%**	**33.3%**	**50.0%**	**0.0%**	**33.3%**	**0.0%**	**100.0%**	**36.4%**	**0.0%**	**100.0%**	**42.9%**	**42.2%**
		*O/W:*	A61P 31/(14-18)	18.8%	16.7%	0.0%	16.7%	50.0%	0.0%	0.0%	0.0%	100.0%	9.1%	0.0%	100.0%	31.4%	32.2%
	A61P 37			6.3%	0.0%	0.0%	0.0%	50.0%	0.0%	0.0%	100.0%	0.0%	0.0%	0.0%	0.0%	34.3%	28.9%
**C12N 27**				**79.2%**	**100.0%**	**100.0%**	**66.7%**	**50.0%**	**88.9%**	**66.7%**	**50.0%**	**100.0%**	**81.8%**	**0.0%**	**100.0%**	**65.7%**	**71.1%**
*Of Which:*	C12N 2760			18.8%	0.0%	0.0%	0.0%	50.0%	88.9%	0.0%	0.0%	0.0%	0.0%	0.0%	0.0%	21.4%	25.0%
	*O/W:*	C12N 2760/16		18.8%	0.0%	0.0%	0.0%	50.0%	88.9%	0.0%	0.0%	0.0%	0.0%	0.0%	0.0%	17.1%	21.1%
	C12N 2770			22.9%	0.0%	100.0%	33.3%	50.0%	0.0%	0.0%	50.0%	100.0%	27.3%	0.0%	0.0%	30.0%	28.3%
	*O/W:*	C12N 2770/20		2.1%	0.0%	0.0%	0.0%	0.0%	0.0%	0.0%	0.0%	0.0%	9.1%	0.0%	0.0%	10.0%	5.0%
	C12N 27xx/xxx23			37.5%	33.3%	100.0%	50.0%	50.0%	66.7%	0.0%	50.0%	100.0%	9.1%	0.0%	0.0%	18.6%	27.8%
	*C12N 27xx/xxx34*			*68.8%*	*100.0%*	*50.0%*	*50.0%*	*50.0%*	*55.6%*	*66.7%*	*50.0%*	*100.0%*	*81.8%*	*0.0%*	*100.0%*	*50.0%*	*58.9%*
**C07K 14**				**66.7%**	**33.3%**	**50.0%**	**16.7%**	**50.0%**	**88.9%**	**66.7%**	**100.0%**	**100.0%**	**100.0%**	**50.0%**	**33.3%**	**61.4%**	**68.9%**
*Of Which:*	C07K 14/(5-19)			50.0%	33.3%	50.0%	0.0%	50.0%	77.8%	0.0%	0.0%	100.0%	90.9%	0.0%	33.3%	45.7%	50.6%
	C07K 14/(195-365)			6.3%	0.0%	0.0%	16.7%	50.0%	0.0%	0.0%	0.0%	0.0%	9.1%	0.0%	0.0%	8.6%	11.1%
	C07K 14/(415-43)			4.2%	16.7%	0.0%	0.0%	0.0%	0.0%	33.3%	0.0%	0.0%	0.0%	0.0%	0.0%	4.3%	5.0%
	C07K 14/(435-4748)			16.7%	0.0%	0.0%	0.0%	0.0%	77.8%	0.0%	0.0%	0.0%	0.0%	50.0%	0.0%	10.0%	11.1%
**C07K 16 & C07K 2317**				**8.3%**	**0.0%**	**0.0%**	**0.0%**	**0.0%**	**0.0%**	**33.3%**	**0.0%**	**50.0%**	**0.0%**	**50.0%**	**33.3%**	**10.0%**	**11.1%**
**C07K 2319**				**27.1%**	**16.7%**	**0.0%**	**16.7%**	**50.0%**	**66.7%**	**33.3%**	**0.0%**	**50.0%**	**18.2%**	**0.0%**	**0.0%**	**32.9%**	**34.4%**
**C12N 9 & C12Y**				**8.3%**	**0.0%**	**0.0%**	**16.7%**	**0.0%**	**0.0%**	**0.0%**	**0.0%**	**100.0%**	**0.0%**	**50.0%**	**0.0%**	**2.9%**	**8.9%**
**C12P**				**10.4%**	**0.0%**	**0.0%**	**16.7%**	**0.0%**	**11.1%**	**33.3%**	**0.0%**	**0.0%**	**0.0%**	**100.0%**	**0.0%**	**1.4%**	**7.8%**
**TOTAL “VACCINE” PATENT FAMILIES (Absolute Values)**				**48**	**6**	**2**	**6**	**2**	**9**	**3**	**2**	**2**	**11**	**2**	**3**	**70**	**180**
***VLP TOTAL* ^[1]^**				***45.2%***	***66.7%***	***100.0%***	***66.7%***	***50.0%***	***66.7%***	***0.0%***	***100.0%***	***100.0%***	***9.1%***	***0.0%***	***33.3%***	***21.4%***	***34.4%***

^[1]^ A61K 2039/5258 *OR* C12N 27xx/xxx23.
